# Changes in the epidemiological characteristics of influenza in children in Zhengzhou, China, in the post-COVID-19 era

**DOI:** 10.1186/s12889-024-19460-3

**Published:** 2024-07-19

**Authors:** Wanyu Jia, Xue Zhang, Ruiyang Sun, Peng Li, Xinggang Zhen, Yu Li, Daobin Wang, Changqing Li, Chunlan Song

**Affiliations:** 1 Henan Province Engineering Research Center of Diagnosis and Treatment of Pediatric Infection and Critical Care, Children’s Hospital Affiliated to Zhengzhou University, Henan Children’s Hospital, Zhengzhou Children’s Hospital, Zhengzhou, 450018 Henan China; 2 Chinese Center for Disease Control and Prevention, BeijingBeijing, 100000 China; 3 Zhecheng County People’s Hospital, Shangqiu, 476200 Henan China; 4 Xinzheng Huaxin People’s Hospital, Zhengzhou, 450000 Henan China; 5 Present Address. : No. 1, South University Road, Erqi District, Zhengzhou, 450018 Henan China

**Keywords:** Influenza, COVID-19, Epidemiological characteristics

## Abstract

**Background:**

Influenza is a contagious respiratory disease posing a huge burden of disease for children around the world. The purpose of this study was to investigate the epidemiologic changes in childhood influenza in Zhengzhou, China, before, during, and after the COVID-19 outbreak. The aim of this study was to determine the impact of the COVID-19 outbreak and related prevention and control policies on the children’s influenza epidemiological trend.

**Methods:**

All influenza report card data from the Children’s Hospital Affiliated with Zhengzhou University’s Disease Surveillance Reporting Management System were collected and analyzed monthly from January 2018 to December 2023. The period of the study was divided into three phases for comparison: the pre-pandemic period, the pandemic period, and the post-pandemic period.

**Results:**

Between January 2018 and December 2023, a total of 82,030 children with influenza were diagnosed at our hospital, including 46,453 males and 35,577 females. A total of 11,833 of them had to be hospitalized for influenza, and 321 of them were brought to the ICU. Influenza showed low-level epidemiologic status during the COVID-19 pandemic, and there was a substantial rise in influenza and a surge in the number of cases after the COVID-19 pandemic period. The year 2023 will had the most influenza cases (40,785). The peak incidence of influenza changes in 2022, from July to October, and in 2023, from February to April and from October to December. During the post-pandemic period, the proportion of new-borns and young children among influenza patients decreased, while the proportion of school-age children increased significantly, and the proportion of influenza patients hospitalized and the proportion of ICU admissions decreased.

**Conclusion:**

Influenza showed low-level epidemiologic status during the COVID-19 pandemic. In the post-pandemic period, there is a large increase in influenza incidence, with a double peak in influenza incidence. The proportion of school-age children with influenza has also increased. As a result, we recommend that influenza vaccination for key populations, particularly school-age children, be completed by October of each year in Henan Province, and that the government and schools increase education about nonpharmacological influenza prevention approaches.

## Background

Influenza is a contagious respiratory disease caused by influenza A and influenza B viruses, which are seasonally prevalent each year. Annual influenza epidemics, according to the World Health Organization (WHO), result in 1 billion infections, 3–5 million cases of severe disease, and 300,000–500,000 deaths [1]. China has included the influenza virus in the national catalog of legally recognized infectious diseases as a Category C infectious disease, which are also known as monitoring and management infectious diseases, patients with such infectious diseases, suspected patients and carriers of infectious diseases required to report should be reported online within 24 h after diagnosis. The population is generally susceptible to influenza viruses, and infants, young children, elderly people, and people with chronic illnesses are at high risk of serious illness and death from influenza [2]. Globally, the incidence of influenza in children under 5 years of age is estimated to be 90 million cases per year [3]. In China, the annual influenza incidence rate among children is 20%-30% during the epidemic season, and the annual infection rate can reach 50% during certain high epidemic seasons [2]. Influenza poses an enormous burden of disease for children around the world. The influenza vaccine is the most effective measure for preventing influenza, and the WHO recommends influenza vaccination for high-risk groups such as children, elderly individuals and medical personnel [4]. The influenza vaccine is a nonimmunization program in China, and residents are vaccinated voluntarily [5]. Therefore, understanding the epidemiological trend of influenza in children can provide a scientific foundation for the creation of efficient preventive and control measures, which are extremely important for the management of epidemics, the reduction of influenza morbidity in children, and the prevention and treatment of epidemics.

In December 2019, COVID-19 began a worldwide pandemic, and in January 2020, China initiated a national blockade in response to the outbreak with emergency measures. Since February 2020, the spread of many respiratory viruses, including influenza viruses, has been significantly suppressed (or decreased), and the incidence of influenza has decreased substantially [6–8]. Between 2020 and 2022, China adopted dynamic zeroing measures for COVID-19 outbreaks which does not require complete “zero infection”, but for each outbreak that occurs, it is required to contain it within a relatively short period of time, before abandoning its three-year zero new crown policy in December 2022 [9]. Subsequently, China lifted preventative and control measures against COVID-19 as a Category A infectious disease on January 8, 2023, with no additional large-scale interventions beginning in January 2023. With the liberalization of the embargo, the population is experiencing mass infections with SARS-CoV-2 Omicron, while cases of other types of infectious diseases are also increasing [9, 10]. Most of the current studies have focused on the decline in the incidence of a wide range of diseases following the COVID-19 outbreak, with fewer studies on the impact on the incidence of a wide range of diseases following the lifting of control measures for the COVID-19 outbreak. The purpose of this study was to investigate the epidemiologic changes in childhood influenza in Zhengzhou, China, before, during, and after the COVID-19 pandemic; to analyze the impact of the COVID-19 pandemic and related prevention and control policies on the epidemiological trend of childhood influenza; and to provide a theoretical basis for the prevention and control of childhood influenza in the future.

## Methods

### Data collection

Our hospital is a large tertiary-level A pediatric specialty hospital with 1810 beds, and a nationally designated sentinel hospital for the surveillance of influenza-like illness in children. Tertiary-level A hospitals are the highest level in the “three levels, six grades” classification of hospitals in mainland China, and are hospitals above the regional level that provide high-level specialized medical and healthcare services and perform higher education and scientific research tasks to several regions. We collected all influenza report card data from the Disease Surveillance Reporting Management System of the hospital from January 2018 to December 2023 and analyzed the data on a monthly basis. The throat swab influenza viral nucleic acid test or influenza antigen test were given to all clinically diagnosed cases with influenza symptoms in our hospital. The Influenza Surveillance Reporting Management System counted all cases of clinically diagnosed influenza in the outpatient and inpatient departments of the hospital with one or more of the following positive pathogenicity tests: [1] positive influenza viral nucleic acid test; (2) positive influenza antigen test.

### Statistical analysis

According to the changes in the COVID-19 pandemic and preventive and control measures, our study period was divided into three main phases, from January 2018 to January 2020 for the pre-pandemic period, from February 2020 to January 2023 for the pandemic period, and from February 2023 to December 2023 for the post-pandemic period. The epidemiologic characteristics of influenza patients in the three periods were then compared. Excel was utilized for data summary processing, while SPSS 26.0 and Excel were used for data analysis and graphing.

## Results

### Characteristics of the study subjects

**Fig. 1:**
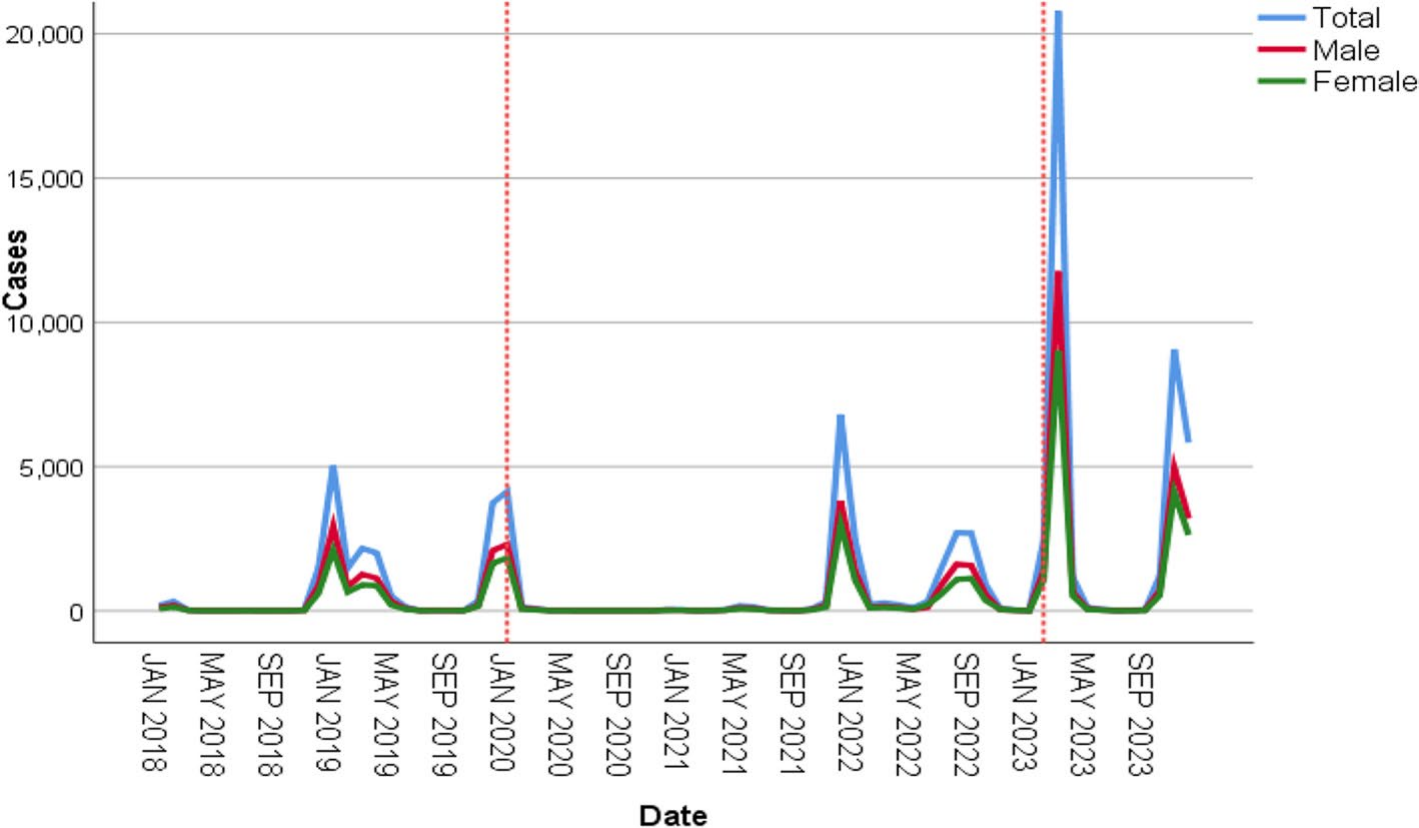
Monthly trends in the number of influenza cases. The dashed lines indicate the point in time when the COVID-19 outbreak began and the COVID-19 pandemic ended

Between January 2018 and December 2023, a total of 82,030 children with influenza were diagnosed at our hospital, including 46,453 males and 35,577 females, and the sex ratio was approximately the same every year, at approximately 1.3:1, with the age ranging from 1 day to 18 years old. Among them, 11,833 children with influenza were in serious condition and required hospitalization, and 321 children were admitted to intensive care units (ICUs) due to critical conditions. The highest number of influenza cases is in 2023 (40,785). (Table 1) Monthly trends in the number of influenza cases over the years are shown in Fig. 1.

**Table 1:** Cases of influenza from January 2018 to December 2023

Year	2018	2019	2020	2021	2022	2023
Total	2130	15,460	4441	7659	11,555	40,785
Male	1255	8864	2492	4310	6706	22,808
Female	875	6596	1949	3349	4849	17,977
Sex ratio	1.43	1.34	1.28	1.29	1.38	1.27
Age group (years)						
≤ 1	473	3443	1312	477	2009	4422
1–3	633	4241	1458	792	3254	7190
4–5	490	3640	959	1559	2943	10,168
≥ 6	534	4136	712	4831	3349	19,005
Hospitalized (%)	424 (19.91%)	4470 (28.91%)	749 (16.87%)	1051 (13.72%)	1438 (12.44%)	3701 (9.07%)
ICU (%)	20 (0.94%)	145 (0.94%)	43 (0.97%)	9 (0.12%)	6 (0.05%)	98 (0.24%)
Death (%)	2(0.094%)	5(0.032%)	5(0.11%)	2(0.026%)	3(0.026%)	20(0.049%)

### Seasonal variation in the number of influenza cases

**Fig. 2:**
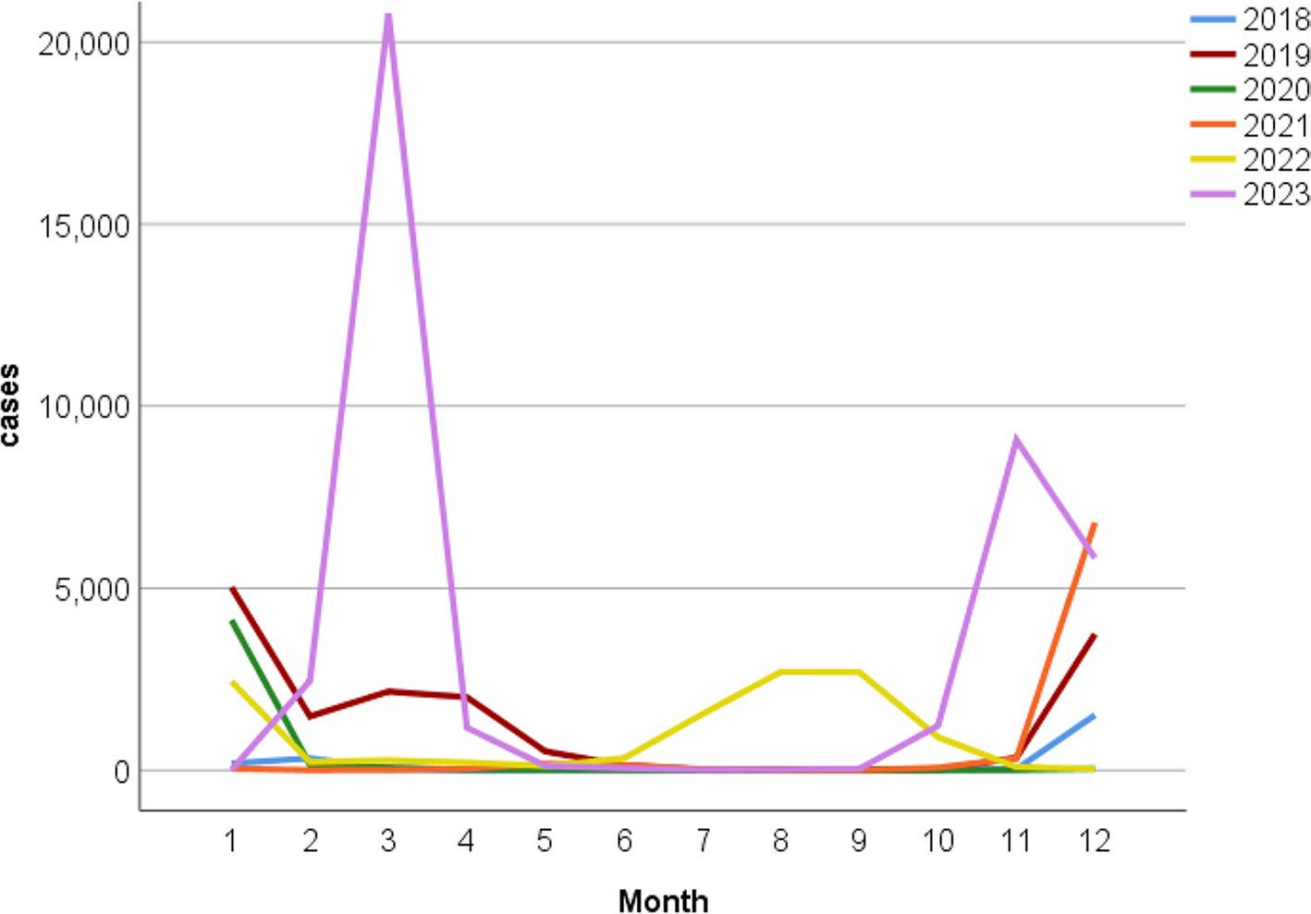
Monthly trends in the number of influenza cases

As shown in Fig. 1 and Fig. 2, in the pre-pandemic period, the peak incidence of influenza was mainly concentrated between November and January. During the pandemic period, after the COVID-19 outbreak and the emergence of state control measures, 2020 saw a significant decrease in the number of influenza cases throughout the year, with no significant peak in the number of influenza cases. In 2021, the number of influenza cases began to increase with the relaxation of state controls and peaked from November through January. In 2022, the number of influenza cases will continue to increase, but the peak incidence will occur from July to October. In the post-pandemic period, there was a substantial increase in the number of influenza cases in 2023 and a change in the peak incidence to February-April and October- December.

### Changes in the age composition of influenza patients

**Fig. 3:**
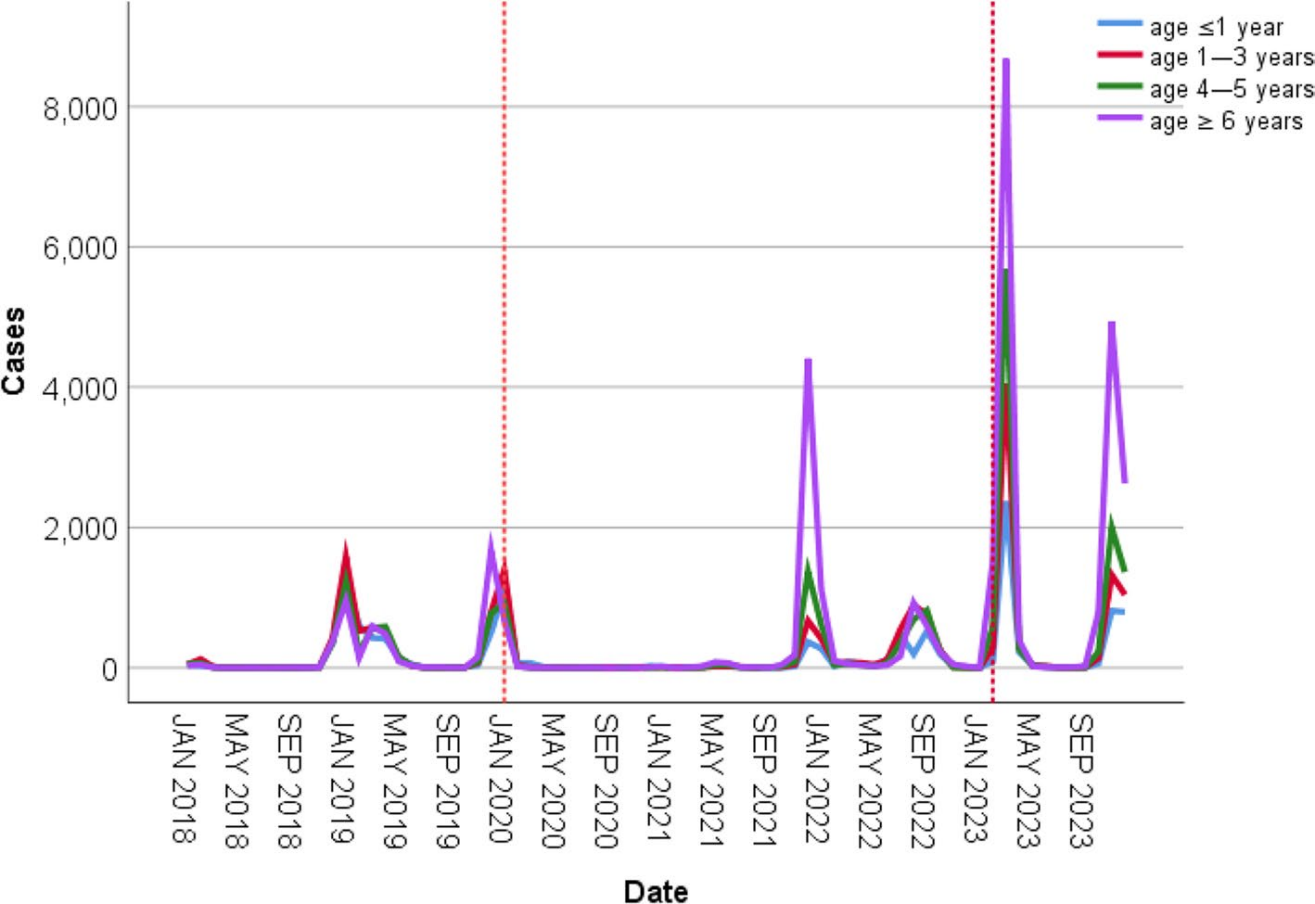
Monthly trends in the number of influenza cases in different age groups. The dashed lines indicate the point in time when the COVID-19 outbreak began and the COVID-19 pandemic ended

**Fig. 4:**
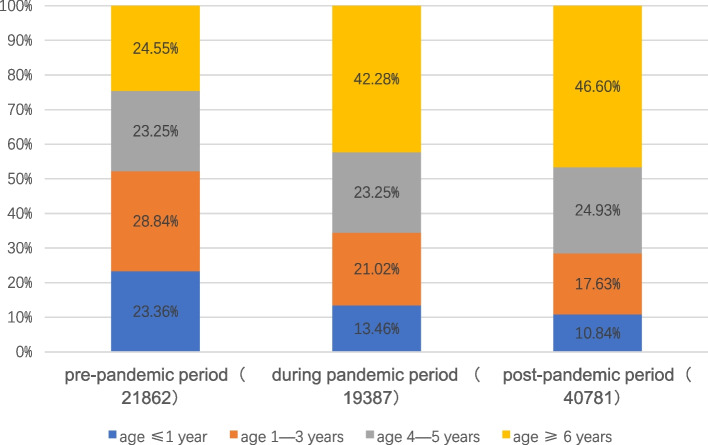
Changes in the age composition of influenza patients. *The numbers in () mean the total number of cases of influenza at different stages. * The horizontal coordinate represents three main phases of our study period

**Fig. 5:**
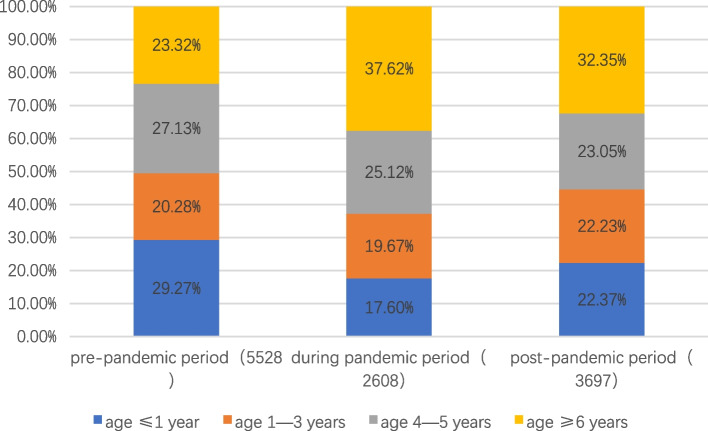
Changes in the age composition of influenza hospitalizations patients. *The numbers in () mean the total number of hospitalizations cases of influenza at different stages. * The horizontal coordinate represents three main phases of our study period

Based on the growth and development of the children, we stratified the patients according to age and divided them into four groups: infancy (less than or equal to 1 year old), early childhood (2–3 years old), preschool (4–5 years old), and school-age (greater than or equal to 6 years old). As shown in Table 1 and Fig. 3, the trend in the number of children with influenza in different age groups was more or less the same as the overall trend. It is interesting to note, however, that following the COVID-19 pandemic, the age distribution of influenza patients shifted. (Fig. 4) In the pre-pandemic period, the majority of influenza cases were in infants under 1 year old and 2–3 years old, with 23.36% and 28.84%, respectively. After the outbreak, there was a decline in the proportion of infants and young children among influenza cases and a significant increase in the proportion of school-age children (6 years and older), which peaked at 46.60%. The trend in age of influenza hospitalizations was consistent with the overall number of cases. (Fig. 5).

### Changes in the proportion of severe and critical influenza cases and deaths

Comparing the pre-pandemic period to the post-pandemic period, we can observe a decrease in the proportion of influenza patients hospitalized and the proportion of ICU admissions. The highest percentage of hospitalized children with influenza was in 2019 (28.91%), and the lowest percentage was in 2023 (9.07%). The proportion of deaths due to influenza did not vary much between the years. (Table 1).

## Discussion

Influenza is a seasonal epidemic that occurs every year. The COVID-19 outbreak and the implementation of national control measures have had an impact on the epidemiological trends of a wide range of diseases [11]. Numerous studies have shown a significant decrease in influenza incidence following the COVID-19 pandemic across various regions of the world [8, 12, 13]. This study analyzed the number of influenza cases in the Children’s Hospital Affiliated with Zhengzhou University between January 2018 and December 2023, and the findings revealed a significant decrease in influenza cases in the first pandemic year following the February 2020 COVID-19 pandemic, followed by a modest increase in the second and third pandemic years. After China’s zero-tolerance policy for COVID-19 was fully lifted in January 2023, there was a surge in the number of influenza cases, which is in general agreement with the findings of Liu P et al. in Shanghai, China [10, 14]. Previously, studies have identified variable increases in influenza, respiratory syncytial virus, and enterovirus during the COVID-19 pandemic (2021–2022) as SARS-CoV-2 infections declined and nonpharmacological interventions were relaxed [15]. Our study similarly confirmed this finding and revealed a more severe backlash against the flu virus in 2023.

This phenomenon may be due to a combination of factors. First, during the pandemic, most children were homebound due to public health control measures and had reduced exposure to influenza viruses, which triggered influenza “immune debt” after prolonged minimal exposure to influenza viruses and decreased immunity in the population [16, 17]. Second, annual influenza vaccination is the most effective means of preventing influenza and significantly reduces the risk of influenza and serious complications for those who are vaccinated [18]. However, due to prolonged home control, most children were not vaccinated against influenza during the pandemic, resulting in the loss of vaccine protection. Moreover, after the lifting of the ZERO-COVID policy in China in December 2022, China experienced a mass infection of the population with the Omicron virus, and although the symptoms of the infection in children were relatively mild, the viral infection was detrimental to the overall immunity of the children; as a result, there was a wave of influenza infections that occurred very soon after the wave of Omicron infections. In addition, this may also be related to changes in patients’ medical treatment behavior during the COVID-19 pandemic. Due to lockdown measures and other reasons, some patients with mild influenza may reduce their visits to hospitals.

Our study similarly revealed that not only did the number of influenza cases change but also the seasonal peaks in the number of influenza cases changed equally during and after the pandemic. The peak number of influenza cases in 2022 and 2023 appears to shift forward and backward to varying degrees. In China, influenza A has a winter epidemic pattern in the northern provinces north of 33 degrees north latitude, a single annual peak in spring in the southernmost provinces south of 27 degrees north latitude, and a double-cycle peak in mid-latitude areas every winter and summer [2]. Henan Province is located in northern China, and the peak season for influenza is from November to January each year. However, in 2022, the peak of the number of influenza cases was changed to July to October, and 2023 showed a double peak epidemic trend, from February to April and from October to December. Meanwhile, it is interesting to note that according to the monthly influenza surveillance report from our Disease control department, influenza A and B were co-prevalent during the peak monthly period of October-December 2023, with influenza B accounting for about one-fourth of the overall total. This was similarly found in a study by Boqiang Chen et al. [19]. This is obviously connected to COVID-19 nonintervention measures. Zhengzhou’s prevention and control measures have gradually loosened since June 2022 due to a decrease in SARS-CoV-2 viral infection, and since October 2022, Zhengzhou has taken highly rigorous control measures due to the large-scale transmission of SARS-CoV-2. This finding reaffirms the impact of nondrug interventions on respiratory transmission diseases and reminds us that in addition to promoting vaccination, nondrug interventions are essential for influenza prevention and control. Therefore, we recommend that children complete their influenza vaccination before October each year in order to provide immune protection.

Another important and interesting finding is that the age composition of influenza cases in children changed during and after the pandemic. In this study, we observed an increasing trend in the prevalence of influenza in school-aged and preschool-aged children. We speculate that this phenomenon may be related to the following: 1. School-aged children are more likely to be exposed to influenza viruses after the pandemic due to school attendance, whereas children under 3 years of age are less likely to be out of the house, and caregivers are more attentive to respiratory viral precautions in the later stages of the pandemic than in the pre-pandemic period, with a decreased likelihood of contracting the virus. 2. A greater proportion of school-aged children are likely to have infections in the January 2023 wave of Omicron infections, and these children may be more susceptible to the influenza virus than are uninfected children for a period of time after infection with Omicron. Dynamic observations are still needed regarding how the age composition ratio of childhood influenza patients changes in the future. Based on the above findings, we suggest that school-age children should be more active in completing influenza vaccination. The government and schools should strengthen the promotion of nonpharmacological interventions for the prevention of respiratory viral infections, such as maintaining good personal hygiene, washing hands frequently, avoiding going to crowded places, and avoiding contact with respiratory infections to maintain good respiratory hygiene practices.

In this study, we also found that there was a decrease in the proportion of hospitalized children and ICU admissions among children with influenza admitted to our hospital. During the pandemic, this may be because nonpharmacologic interventions not only reduce the spread of influenza but also reduce the likelihood of severe illness. In the post-pandemic, even we think that children’s immunity to influenza generally declines, but we found that although the number of children with influenza increased, the proportion of children hospitalized with influenza declined. The decrease in the proportion may be attributed to the fact that more parents refused to be hospitalized in the aftermath of the pandemic due to concerns such as cross-infection. Another reason may be that the number of cases soared due to the limited number of beds in hospitals, preventing some of the children who needed to be hospitalized from being admitted to the hospitals. This may also be because after the COVID-19 pandemic, people’s awareness and attention to infectious diseases increased, so treatment of the disease in the early stage was more active, preventing the development of severe disease. However, the current observation period is relatively short, and long-term observation is still needed for future changes in the proportion of severe childhood influenza cases.

In summary, our study revealed that the COVID-19 pandemic and nonpharmacological interventions had dramatic impacts on influenza epidemics in terms of the number of influenza cases, peak, age composition, and proportion of critical illnesses. In the post-pandemic period, there was an outbreak and a forward shift in the peak incidence of influenza cases due to several factors, including immunization debt, which reminds us to pay attention not only to the spread of SARS-CoV-2 after the deregulation of COVID-19 but also to the pandemics of other types of respiratory transmissible diseases.

Our study has some limitations. We examined the medical files from only one hospital. And due to the absence of records in the case system, we do not have access to the immunization coverage of the catchment population and the immunization coverage of the patient population over the study period. However, the number of cases is representative of China’s largest tertiary-level A pediatric specialty hospital and a nationally designated sentinel hospital for the surveillance of influenza-like illness. Our observation of changes in the number of influenza cases in the post-pandemic period was short-lived, and longer monitoring will be needed in the future. Since we did not have access to influenza type information, we analyzed only the overall cases of influenza without grouping them according to the type of influenza virus, but the seasonal epidemics of influenza are mainly dominated by influenza A; therefore, the results of this study are still informative. Meanwhile, according to the 2023 influenza detection report of the Department of Disease Control and Prevention of our hospital, the influenza epidemic in the peak period from February to April of 2023 was dominated by influenza A, and the incidence of influenza B was less. The peak period from October to December in 2023 showed a common epidemic trend of influenza A and influenza B, and influenza B accounted for about 1/4 of the total, which showed an increasing trend compared with February-April.

## Conclusion

In conclusion, the epidemiologic pattern of influenza in China changed both during the COVID-19 pandemic and in the latter part of the pandemic. There was a surge in the number of influenza cases in the latter part of the pandemic, along with an earlier peak of influenza onset and an increase in the proportion of school-aged children with influenza. Therefore, we recommend that influenza vaccination for key populations, especially school-age children, be completed by October each year, and that the government and schools increase education on nonpharmacological interventions to prevent influenza.

## Data Availability

The datasets used and/or analysed during the current study are available from the corresponding author on reasonable request.

## Abbreviation

ICU: Intensive care units
WHO: World Health Organization
